# Immediate postpartum anemia and associated factors among women admitted to maternity ward at public hospitals in Harari Regional State, Eastern Ethiopia: A facility-based cross-sectional study

**DOI:** 10.3389/fgwh.2022.916245

**Published:** 2022-09-20

**Authors:** Gizaw Taddesse Abebe, Mohammed Abdurke Kure, Tesfaye Assebe Yadeta, Kedir Teji Roba, Tariku Dingeta Amante

**Affiliations:** ^1^Department of Midwifery, College of Health and Medical Sciences, Dilla University, Dilla, Ethiopia; ^2^School of Nursing and Midwifery, College of Health and Medical Sciences, Haramaya University, Harar, Ethiopia; ^3^School of Public Health, College of Health and Medical Science, Haramaya University, Harar, Ethiopia

**Keywords:** anemia, postpartum, associated factors, hemoglobin, Ethiopia, immediate postpartum

## Abstract

**Background:**

Anemia in the postpartum period remains a considerable public health problem in developing countries, particularly in sub-Saharan Africa. It is the most common indirect cause of maternal morbidity and mortality. It is also a major health problem in women of reproductive age, affecting their quality of life, occupational capacity, lactation, and immunological function. Immediate postpartum anemia has a significant impact on women's quality of life, although its predictors have received little attention in Ethiopia, notably in Harari Regional State. Therefore, this study aimed to determine its magnitude and contributing factors in Eastern Ethiopia.

**Methods:**

A facility-based cross-sectional study was conducted from June 1^st^ to August 30^th^, 2021, among 484 postpartum women admitted to two public hospitals in Harari Regional State, Eastern Ethiopia. Data were collected using a pre-tested, structured interviewer-administered questionnaire. About 2 mL of blood samples were collected and analyzed using the cell-Dyne 1,800 machine. The collected data were entered into Epi-Data version 4.6 and analyzed using SPSS version 25. A multivariable logistic regression analysis was conducted to estimate the effect of independent variables on immediate postpartum anemia. An adjusted odds ratio (AOR) with a 95% confidence interval (CI) was computed to report the presence of the association. Statistical significance was declared at a *p-*value of < 0.05.

**Results:**

The overall magnitude of immediate postpartum anemia was 28.1% [95% CI (23.7, 32.1)]. Lack of formal education [AOR: 3.92; 95% CI: (1.85, 8.33)], having antenatal care < 4 visits [AOR: 3.18; 95% CI: (1.53, 6.61)], a history of cesarean delivery [AOR: 3.40; 95% CI: (1.89, 6.10)], a history of maternal blood loss [AOR: 4.78; 95% CI: (2.22, 10.30)], pre-delivery Hgb level < 11 g/dl [AOR:5.46; 95% CI: (3.09,9.67)], and having no iron-folate supplementation [AOR:3.27; 95% CI: (1.31, 8.15)] were factors statistically associated with immediate postpartum anemia.

**Conclusions:**

In this study, nearly one-third of mothers admitted for postpartum care developed anemia within 48 h of giving birth. Women's educational level, frequency of antenatal care, mode of delivery, a history of maternal blood loss, pre-delivery hemoglobin level, and iron-folate supplementation status were identified as immediate postpartum anemia risk factors. Therefore, promoting the benefits of adequate antenatal care and iron-folate supplementation during pregnancy is crucial to avoiding the risks of postpartum anemia.

## Introduction

Anemia is a condition characterized by a reduction in the number and/or size of red blood cells below cut-off values, thereby impairing the blood's ability to transport oxygen to meet physiologic needs (1). Even though postpartum anemia (PPA) lacks a consensual definition, it can be inferred from the definitions provided by various scholars, depending on the duration of the postpartum period. It can be defined as Hgb < 10 g/dl, Hgb < 11g/dl, and Hgb < 12g/dl cut-off values within the first 48 h of delivery, at 1 week and 6 weeks of postpartum duration, respectively (2–5).

Although hopeful progress has been made in lowering maternal mortality in many countries, there is still evidence of a continuous increment in the rate of indirect causes of maternal mortality (6). Globally, indirect causes of maternal mortality are attributable to 35% of all causes of maternal deaths, with anemia accounting for 7% of maternal mortality due to indirect causes and 2.3% of all causes (7, 8). Moreover, severe postpartum anemia increases the risk of maternal mortality by 3 folds during the postpartum period (9).

In 2019, anemia caused 1.74 billion cases and 58.6 million years of disability worldwide (10). More specifically, PPA affects 50–80% of postpartum women in developing countries and less than 30% in developed countries (7, 11). Approximately 36.5% of lactating women were particularly vulnerable to PPA in East African countries (12). The prevalence of PPA during lactation in Ethiopia also ranges from 11.6% in Addis Ababa to 58.7% in the Somali Region (13).

Furthermore, researchers have shown that postpartum anemia (PPA) has been found to be one of the most common causes of morbidity in the early postpartum period. In addition, it is also a major health problem in women of reproductive age, affecting their quality of life, occupational capacity, lactation, and immunological function (11, 14, 15). Additionally, PPA has been strongly associated with postpartum depression and emotional instability, negatively affecting mother-infant bonding (16, 17). This implies alterations during the child's psycho-neurological development, which can negatively affect infant development (18).

Studies have revealed that socio-demographic factors, obstetric-related factors, and nutritional and maternal health status are associated with PPA. For instance, various research reports have found that the age of the mother (19, 20), area of residence (21), level of education (21, 22), history of maternal blood loss (23, 24), pre-delivery Hgb level (23, 25), history of episiotomy during childbirth (26), mode of delivery (27), number of antenatal care visits, maternal parity (13), maternal nutritional status (24), history of iron and folic acid supplementation (28), and household food insecurity status (29) are significantly associated with PPA. Moreover, chronic medical illnesses such as malaria and HIV (28) are also associated with an increased risk of PPA.

In Ethiopia, although few studies have been conducted in the last decade, almost all previous studies focused on assessing lactating women after they were discharged from the facility (29–31), which may delay the testing of PPA, resulting in logistical challenges and late initiation of the treatment, finally resulting in short- and long-term complications of anemia (15, 32). Moreover, although the maternity ward is found to be a favorable place for the diagnosis of anemia, approximately 64% of women in Ethiopia received postpartum care late (33). Therefore, this study aimed to assess the magnitude of immediate postpartum anemia and its contributing factors among postpartum women admitted to maternity wards within 48 h of delivery in public hospitals in Harari Regional State, Eastern Ethiopia.

## Methods and materials

### Study setting and design

A facility-based cross-sectional study was conducted from June 1^st^ to August 30^th^, 2021, in two public hospitals in Harari Regional State, Eastern Ethiopia. The Harari region is one of the 11 regions in Ethiopia, which are found in Eastern Ethiopia. Harar is the capital city of the Harari Regional State, located 526 km east of the capital city, Addis Ababa. According to the Ethiopian census projection for 2019/2020, the region has an estimated population of 263,656, including 60,667 reproductive-age women (34). There are two public hospitals, one private hospital, one police hospital, eight health centers, 54 private clinics, and 24 health posts in the region.

The study was conducted at Hiwot Fana Comprehensive Specialized Hospital (HFCSH) and Jugal Regional Hospital (JRH). HFCSH is the tertiary health care level (a referral hospital hosted by Haramaya University), and Jugal Regional Hospital (JRH) is the only regional hospital in Harari Regional State. Both hospitals currently provide comprehensive care for more than five million people in their catchment area. Hiwot Fana Comprehensive Specialized Hospital (HFCSH) and Jugal Regional Hospital (JRH) have 60 and 23 beds in the maternity ward, respectively. According to Health Management and Information System (HMIS), the estimated annual maternal delivery services of HFCSH was 4,680 (35), and JRH was 3,204 (36).

### Population, eligibility criteria, and sampling procedure

In this study, all postpartum women admitted to the maternity wards of two public hospitals (HFCSH and JRH) were enrolled within the first 48 h of delivery. However, women who delivered by cesarean hysterectomy or laparotomy after uterine rupture and multiple pregnancies were excluded to reduce the overestimation of immediate postpartum anemia. Moreover, all postpartum women with the following preconditions (had known anemia before conception, transfused blood in the intrapartum and postpartum period, and were critically ill during the data collection period) were excluded from the study.

In this study, the sample size was calculated by using statistical software Epi-info version 7.2.4.0 (the USA, 2018) by considering the following assumptions: A proportion of immediate postpartum anemia (*p* = 24.2%) taken from a study conducted at Mekele, Ethiopia (21), a 4% tolerable margin of error (d = 0.04), a 95% confidence level (Z_α/2_ = 1.96), and 10% contingency for non-response rate. The final sample size for this study was 484.

**Figure 1:**
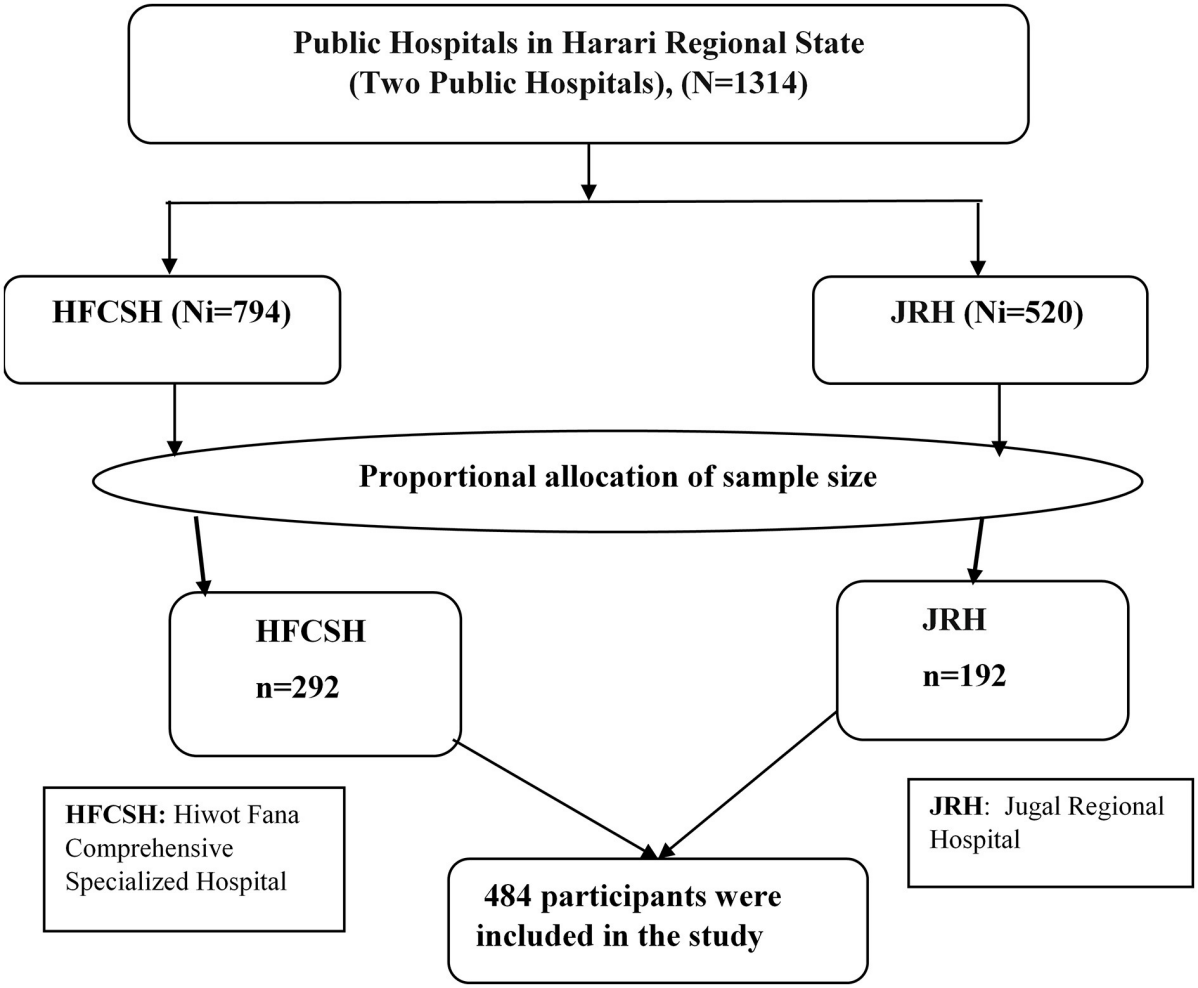
Schematic presentation of sampling procedure to select immediate postpartum women admitted to the maternity ward in public hospitals in Harari Regional State, Eastern Ethiopia, 2021.

A systematic sampling technique was used to select a total of 484 study participants. Initially, the only two public hospitals in Harari Regional State were selected purposely as they provided delivery services in the region. According to the previous information obtained from the Health Management and Information System (HMIS) report (6-month report), 2,380 and 1,560 women were admitted to the maternity wards of HFCSH and JGH, respectively, for postnatal care services, including those who gave birth at home or other health facilities. The data collection period was adjusted for 2 months to get the required sample size. Therefore, the total number of the 6-month report was divided into three to get 2 months' report. Thus, the total number of 2-month postpartum women at both hospitals became 1,314. Proportionally, we allocated 794 and 520 women to HFCSH and JRH, respectively. Then, the total sample size (*n* = 484) was proportionally allocated to 292 and 192 women from HFCSH and GRH, respectively. We also calculated the K^th^ interval for both hospitals (K^th^ = 1314/484 ≈ 3). Therefore, every third eligible postpartum woman was selected and interviewed until the required samples were obtained (Figure 1).

### Data collection tools and procedures

The data were collected using a pre-tested structured and interviewer-administered questionnaire adopted and customized from different kinds of literature (13, 21, 24, 37). Reviews of maternal charts were also undertaken using validated checklists. The data were collected over-period of 2 months by six trained data collectors (four new graduates of bachelor of science (BSc) degree midwives and two bachelor of sciences (BSc) degrees in medical laboratory scientists). In addition, two masters of midwives were recruited for the supervision of data collectors and the data collection process.

In this study, the questionnaire for maternal information contains four main parts: socio-demographic factors, obstetric-related factors, nutritional-related factors, and comorbid disease-related factors. Food insecurity status was measured using the Household Food Insecurity Access Scale (HFIAS), which was recommended by food and nutrition technical assistance (FANTA) to stratify individuals as food secure or food insecure (38). It is valid and reliable in Ethiopia as measured by Cronbach's alpha value of 0.85 for both rural and urban samples (39). Mid-Upper Arm Circumference (MUAC) was measured using tape measures on non-dominant hands, and the result was interpreted to the UNICEF and WHO recommendation cut-off point.

A combination of data collection methods was used. The data from mothers were collected using a pre-tested structured and face-to-face interviewer-administered questionnaire with reviews of maternal charts for clarity of diagnosis and intervention. The data collection tool for maternal (40) was prepared first in English, translated into a local language (Afan Oromo and Amharic), and then re-translated back to English by the experts.

Initially, all medical records/charts of mothers were screened for the presence of any diagnosed medical and/or obstetrical complications, pre-delivery Hgb level, newborn weight, and other clinical characteristics. The laboratory technician collected approximately 2 mL of blood from each study participant, especially after 8 h of delivery. Hgb concentration was determined using the automated blood analyzer Cell-Dyne1800 (Abbott Laboratories Diagnostic Division, USA) by the laboratory technician. Hemoglobin levels were collected and attached to the participants' folders before they were discharged from the hospitals. Finally, anemic mothers were managed based on their Hgb levels according to national guidelines with IFA or blood transfusion and counseled on iron-rich diet intake after being informed by the focal person of the unit (ward).

### Variables and measurements

#### Outcome variable

An outcome variable was immediate postpartum anemia. If a woman's Hgb level was <10 g/dl, it was considered to be anemic (yes), and if the Hgb level was 10 g/dl or above, it was considered non-anemic (no) (24). We recorded this dependent variable during the analysis stage as the binary outcome of 0 and 1. Thus, if the woman's Hgb level was <10 g/dl, it was recorded as 1; if the Hgb level was ≥10 g/dl, it was recorded as 0.

### Independent variables

In this study, the explanatory variables include socio-demographic-related characteristics (maternal age, place of residence, education level, occupation, and monthly income of the family), obstetrics or reproductive-related factors (parity, ANC visits, time of ANC contacts, mode of delivery, birth weight of the newborn, episiotomy, history of maternal blood loss, pre-delivery Hgb level, birth interval, intrapartum and postpartum complications, medical conditions or chronic disease-related factors (malaria, HIV positive, medical diseases, intestinal infestation), and nutritional related factors (frequency of meals and levels of MUAC measurement).

#### Measurements

In this study, adherence to iron supplementation is said to be good if the mother took iron supplementation for ≥3 months but poor if taken for less than 3 months during the most recent pregnancy) (41, 42), and **food insecurity** is a lack of consistent access to a sufficient amount of healthy, nutritious, and culturally appropriate food for every person in a household due to a lack of money and other resources to live an active, healthy life. It is measured by the Household Food Insecurity Access Scale (HFIAS) based on nine occurrence questions developed by NATA and validated in Ethiopia (43). It is classified as food secure if the women scored two or fewer affirmative (yes) answers and food insecure if they scored more than two affirmative (yes) answers.

### Data quality control

Structured and pre-tested tools were used. The final version of the questionnaire for maternal information was translated from the English language into the local language by experts. The training was given to data collectors and supervisors. A pre-test was conducted on 5% of the sample size at another public hospital (40) with similar characteristics to the study population. Any ambiguity, confusion, and difficult words were removed and verified based on pretested experience. A continuum of close supervision was ensured on each data collection day. Two independent data clerks did double data entry.

### Data processing and analysis

The collected data were coded, cleaned, and entered into Epi-Data version 4.6 and then exported to SPSS version 25 (IBM SPSS Statistics, 2016) for further analysis. Descriptive statistics were carried out using frequency tables, proportions, and summary measures. Bivariable and multivariable logistic regression analyses with a 95% Confidence Interval (CI) were used to identify factors independently associated with immediate PPA. From the bi-variable analysis, variables with a significant level of *p*-value < 0.25 were considered for the multivariable analysis model to control for potential confounders. During multivariable analysis, the model fitness was checked by the Hosmer-Lemeshow model fitness test, and the result was insignificant (p = 0.828). Moreover, multi-collinearity was also checked using the variance inflation factor (all VIF values were < 2.8) and standard error < 2. Finally, an adjusted odds ratio (AOR) with a 95% CI was used to indicate the relationship between the independent variables and the immediate postpartum anemia. The statistical significance was declared at a *p-*value less than 0.05.

### Ethical considerations

The study protocol was approved by the Institutional Health Research Ethics Review Committee (IHRERC) of the College of Health and Medical Sciences, Haramaya University (IHRERC/091/2021). All participants were informed about the procedure and purpose of the study, and informed, written, and signed voluntary consent was obtained before participation. Additionally, informed, written, and signed voluntary assent was taken from the parent, husband, or guardian using normal disclosure processes for participants less than 18 years of age. Other additional data needed were extracted from medical records; confidential information about the identity of individual patients was not collected. The standard safety measures for the prevention and transmission control measures of COVID-19 were strictly followed and carried out during the data collection period as per the WHO 2021 standard.

## Results

### Socio-demographic characteristics of the study participants

A total of 477 selected postnatal women were enrolled in this study, yielding a response rate of 98.6%. The mean age of the study participants was 25.33 (*SD*± 5.24) years. Nearly one-third of the study participants, 134 (28.1%), had no formal education. Slightly more than half (51.8%) of the participants' income was less than 2,500 ETB, with a median income of 2,500 ETB and IQ ± 2500 ETB (Table 1).

**Table 1:** Socio-demographic characteristics of postpartum women admitted to the maternity ward in public hospitals in Harari Regional State, Eastern Ethiopia, 2021 (*n* = 477).

**Characteristics**	**Categories**	**Frequency (n)**	**Percentage (%)**
Age of the mother (years)	15–24	216	45.3
	25–34	234	49.0
	35–49	27	5.7
Current residence of the mother	Rural	277	58.1
	Urban	200	41.9
Marital status of the mother	Married	460	96.4
	Divorced	10	2.1
	Others*	7	1.5
The religion of the mother	Orthodox	68	14.3
	Muslim	362	75.9
	Protestant	47	9.9
Maternal educational level	Lack formal education	134	28.1
	Primary school (1-8)	184	38.6
	Secondary (9-12) and above	159	33.3
Occupational status	Housewife	312	65.4
	Government employee	41	8.6
	Private employee	38	8.0
	Merchant	86	18.0
Husband educational level	No formal education	82	17.8
	Primary school	179	38.8
	Secondary and above	200	43.4
Husband occupation	Non employed	11	2.4
	Government employer	95	20.6
	Private employee	98	21.3
	Merchant	115	24.9
	Farmer	142	38.8
Average monthly income of the family(in ETB/USD)	<1000ETB (<10USD)	75	15.7
	1000–3000ETB (10–60USD)	248	52.0
	3001–5000ETB (60.02–100USD)	119	24.9
	≥5001ETB (≥100.02 USD)	35	7.3

ETB, Ethiopian Birr; USD, United State Dollar; ^*^, Widowed or separated.

### Obstetric-related characteristics of the study participants

The majority of the participants, 252 (52.8%), were multiparous, and 126 (65.8%) had short inter-pregnancy intervals (<2 years). More than half (56.7%) of the participants had <4 ANC visits, and about one-eighth (11.9%) of the participants had a history of maternal blood loss in recent pregnancy (Table 2).

**Table 2:** Obstetrics-related factors of postpartum women admitted to the maternity ward in the public hospitals in Harari regional state, Eastern Ethiopia, 2021.

**Characteristics**	**Categories**	**Frequency(n)**	**Percentage (%)**
Parity	Primiparous	130	27.3
	Multiparous	252	52.8
	Grand multiparous	95	19.9
History of abortion	Yes	62	13
	No	415	87
Inter-pregnancy interval in year	<2	121	34.2
	≥2	233	65.8
Current ANC visits	Yes	356	74.6
	No	121	25.4
GA at first ANC initiation	<16 weeks	145	40.7
	≥16 weeks	211	59.3
Frequency of ANC visits	<4 times	202	56.7
	>4 times	154	43.3
Maternal blood loss	Yes	57	11.9
	No	420	88.1
Place of delivery	Health facility	471	98.7
	Others	6	1.3
Gestational age at delivery	Preterm (<37 weeks)	122	25.6
	*Term*≥(37 weeks)	355	74.4
Mode of delivery	SVD	336	70.4
	IAVD	25	5.2
	C/S	116	24.3
Manual removal of placenta	Yes	5	1.0
	No	472	99.0
Episiotomy at current delivery	Yes	53	11.1
	No	424	88.9
Prolonged 2^nd^ stage of labor	Yes	98	20.5
	No	379	79.5
Perineal tear/laceration	Yes	35	7.3
	No	442	92.7
Weight of newborn in grams	<2,500	64	13.9
	2,500–3,999	329	69.0
	>4,000	84	17.6
Pre-delivery hemoglobin level	<11 g/dl	113	23.9
	≥11 g/dl	359	76.1

IAVD, instrumental assisted vaginal delivery; C/S, cesarean-section; SVD, Spontaneous vaginal delivery.

### Comorbid disorders-related characteristics

**Figure 2:**
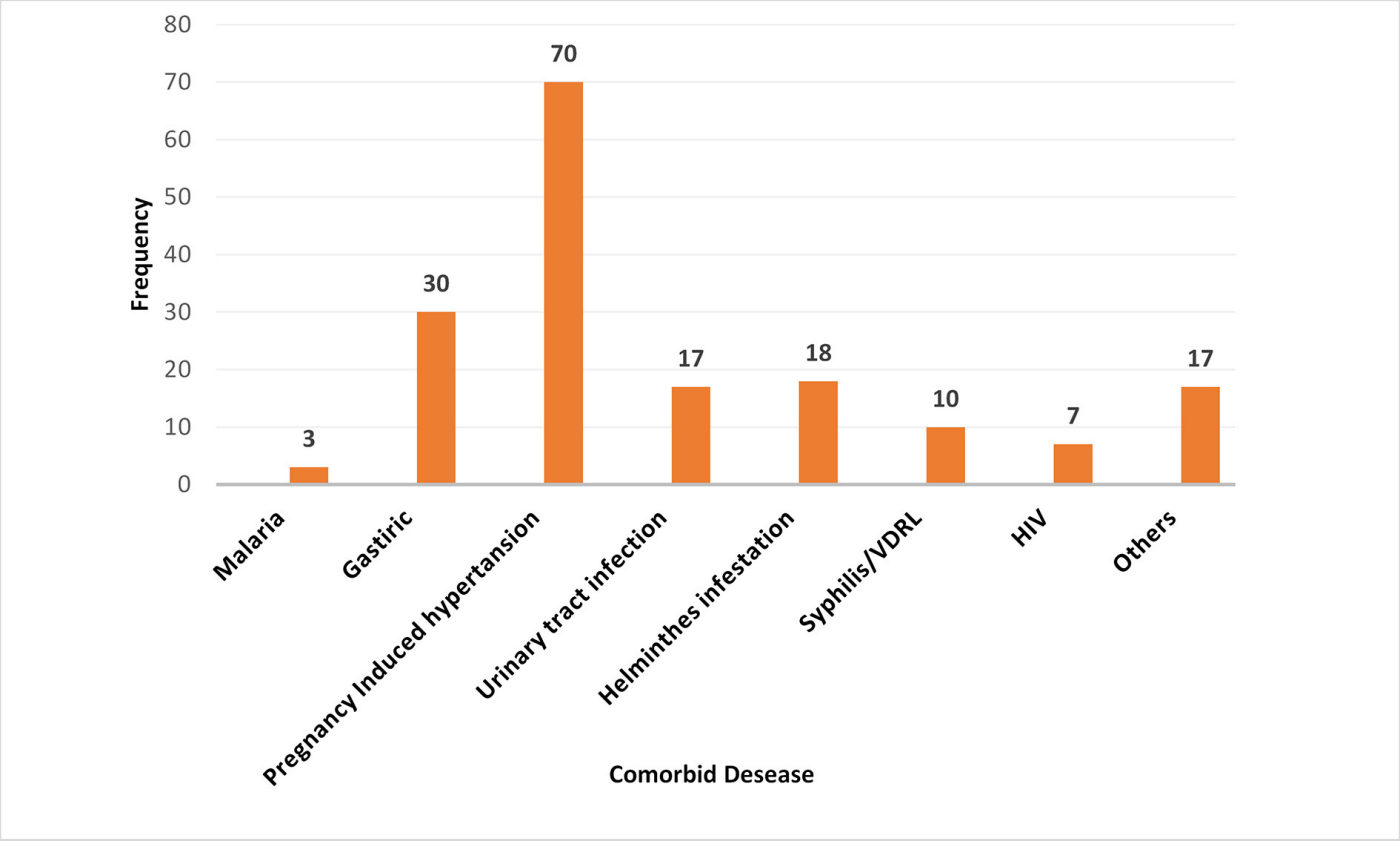
Comorbid disorders during pregnancy among postpartum women admitted to the maternity ward in public hospitals in Harari Regional State, Eastern Ethiopia, 2021. Other: Diabetes Mellitus, Tuberculosis, Pre-conception anemia, Heart failure, chronic hypertension.

Among all study participants, 132 (27.7%) had clinical or diagnostic tests that confirmed medical and/or obstetric complications during recent pregnancy. Pregnancy-induced hypertension was the most reported condition (14.8%), whereas a history of malaria was the most minor medical disease reported in this study (0.6%) (Figure 2).

### Dietary diversity and micronutrient-related characteristics

In this study, nearly three-fourths (73.8%) of the study participants started iron-folate during the most recent pregnancy, and more than half (57.4%) of them had poor adherence to iron-folate. The majority, 397 (83.2%), of the study participants' mid-upper arm circumference was 23 cm or above (Table 3).

**Table 3:** Dietary and micronutrient-related factors of postpartum women admitted to the maternity ward in public hospitals in Harari Regional State, Eastern Ethiopia, 2021.

**Characteristics**	**Categories**	**Frequency(n)**	**Percentage (%)**
Utilized IFA during the current pregnancy	Yes	352	73.8
	No	125	26.2
Time of first IFA initiation during current pregnancy	<16 weeks	140	49.8
	20–24 weeks	129	36.6
	26–30 weeks	66	18.8
	30–34 weeks	17	4.8
Level of adherence to IFA tablets	Poor	202	57.4
	Good	150	42.6
Utilizing hot drinks with IFA	Yes	105	29.8
	No	247	70.2
Type of utilized hot drinks	Tea	15	14.3
	Coffee	80	76.2
	Milk	10	9.5
Frequency of meal/day	<3 times	113	23.7
	≥3 times	364	76.3
Food insecure in household	Yes	51	10.7
	No	426	89.3
Maternal MUAC measurement	≥23 cm	397	83.2
	<23 cm	80	16.8

IFA, Iron-folic Acid; GA, gestational age; MUAC, middle upper arm circumference.

### The magnitude of immediate postpartum anemia

**Figure 3:**
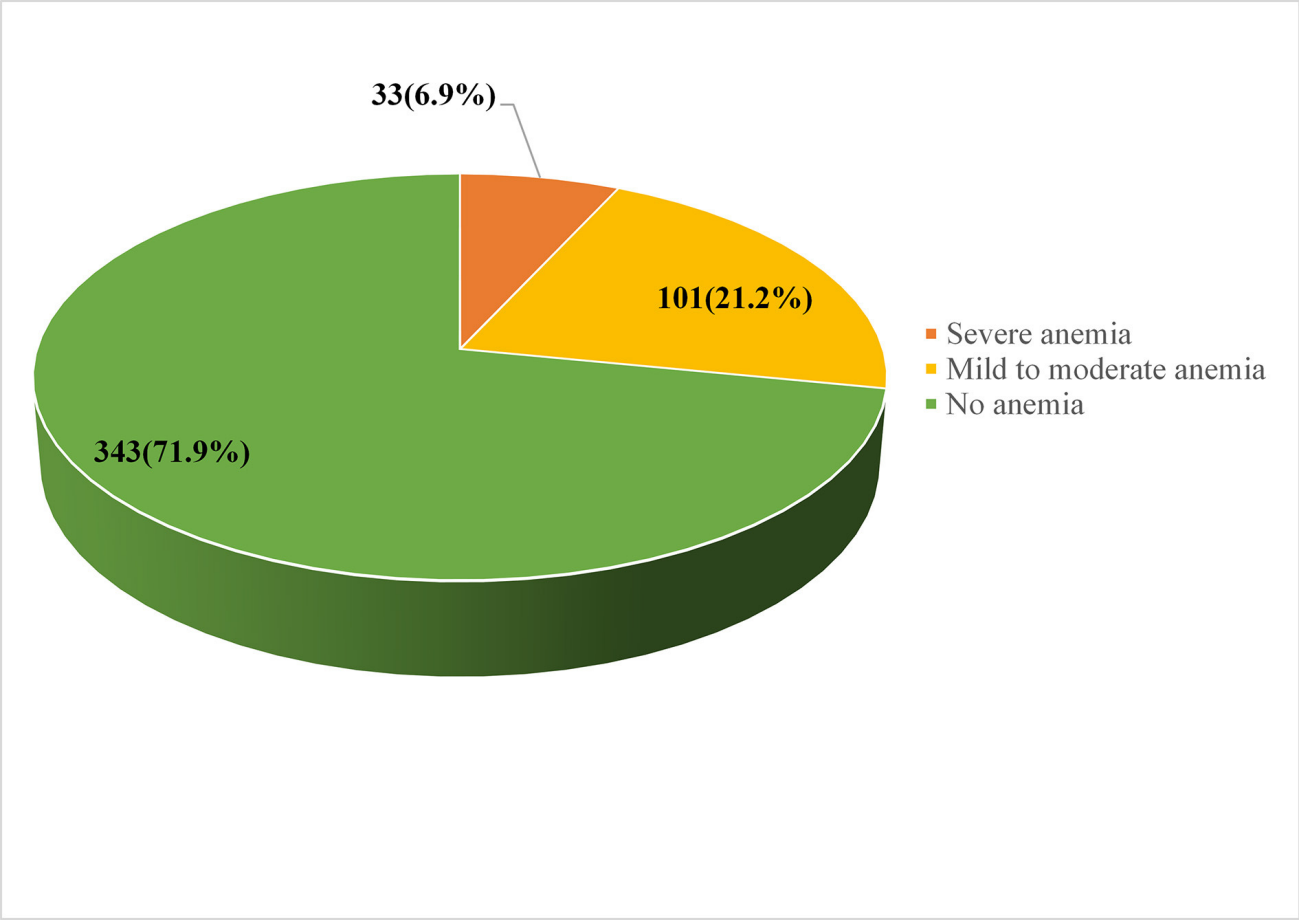
Magnitude of immediate postpartum anemia among women admitted to the maternity ward in public hospitals in Harari Regional State, Eastern Ethiopia, 2021.

The overall magnitude of immediate postpartum anemia was 28.1% [95% CI (23.7–32.1%)]. The postpartum hemoglobin concentration of the participants ranged from 3 g/dl to 14.8 g/dl, with median values of ± 10.5 g/dl (Figure 3).

### Factors associated with immediate postpartum anemia

In bivariable analysis, mothers' age, place of residence, educational level, maternal parity, inter-pregnancy interval, frequency of ANC follow-up, mode of delivery, gestational age at delivery, a history of maternal blood loss, pre-delivery Hgb level, iron-folate supplementation, and MUAC level of the mothers were independently associated with immediate postpartum anemia among postpartum mothers. However, in the final model of multivariable logistic regression analysis, the educational level of the mother, frequency of ANC follow-up, mode of delivery, a history of maternal blood loss, pre-delivery Hgb level, and IFA supplementation during recent pregnancy were factors that remained statistically associated with immediate postpartum anemia.

Accordingly, the likelihood of immediate PPA was four times higher among women with no formal education than among those with at least secondary education or above [AOR: 3.92; 95% CI (1.85, 8.33)]. Likewise, women who had fewer than four ANC visits were three times more likely to be anemic than those who had at least ≥ 4 ANC visits during their current pregnancy [AOR: 3.18; 95% CI (1.53, 6.61)]. Similarly, the odds of immediate PPA were three times higher among women who gave birth through cesarean section (C/S) than among those who delivered vaginally [AOR: 3.40; 95% CI (1.89, 6.10)]. Moreover, the likelihood of immediate PPA was nearly five times higher among women with a history of blood loss than their counterparts [AOR: 4.78; 95% CI (2.22, 10.30)]. Likewise, women with a pre-delivery Hgb level of <11 g/dl were 5.5 times more likely to be anemic than those whose Hgb levels were ≥ 11 gm/dl [AOR: 5.46; 95% CI (3.09, 9.67)]. Furthermore, women with no history of iron-folate supplementation during their current pregnancy were three times more likely [AOR: 3.27; 95% CI: (1.31, 8.15)] to be anemic than those with a history of iron-folate supplementation during their most recent pregnancy (Table 4).

**Table 4:** Bi-variable and multivariable analysis of factors associated with immediate postpartum anemia among postpartum women admitted to the maternity ward in public hospitals in Harari Regional State, Eastern Ethiopia, 2021 (N = 477).

**Factors**	**Categories**	**Immediate PPA**		**COR (95%CI)**	**AOR (95%CI**	***p*–value at AOR**
		**Yes (%)**	**No (%)**			
Age of the mother (years)	15–24 years	57 (26.4)	159 (73.6)	1	1	
	25–34 years	61 (26.1)	173 (73.9)	0.98 (0.65–1.50)	0.93 (0.47–1.82)	0.826
	35–49 years	16 (59.3)	11 (40.7)	4.06 (1.78–9.26)	3.36 (0.99–11.49)	0.050
The educational level of the mother	Lack of formal education	70 (52.2)	64 (47.8)	6.47 (3.71–11.29)	**3.92 (1.85–8.33)**	0.0001
	Primary (1–8)	41 (22.3)	143 (77.7)	1.70 (0.97–2.97)	1.35 (0.68–2.71)	0.390
	Secondary (9–12) and above	23 (14.5)	136 (85.5)	1	1	
Residence of the mother	Rural	101 (36.5)	176 (63.5)	2.90 (1.86–4.54)	1.40 (0.73–2.66)	0.310
	Urban	33 (16.5)	167 (83.5)	1	1	
Parity of the mother	Primipara	32 (24.6)	98 (75.4)	1	1	
	Multiparous	64 (25.4)	188 (74.6)	1.04 (0.64–1.70)	0.63 (0.12–3.22)	0.574
	Grand–multiparous	38 (40.0)	57 (60.0)	2.04 (1.15–3.62)	0.43 (0.07–2.50)	0.346
Frequency of ANC visits	<4 visit	73 (36.1)	129 (63.9)	4.88 (2.70–8.82)	**3.18 (1.53–6.61)**	0.002
	**≥**4 visit	16 (10.4)	138 (89.6)	1	1	
Inter–pregnancy interval	<2 years	43 (35.5)	78 (64.5)	1.49 (0.93–2.38)	1.27 (0.69–2.33)	0.451
	**≥**2 years	63 (27.0)	170 (73.0)	1	1	
Mode of delivery	SVD	70 (20.8)	266 (79.2)	1	1	
	IAVD	9 (36.0)	16 (64.0)	2.14 (0.91–5.04)	1.97 (0.64–6.09)	0.238
	C/S	55	61	3.43 (2.19–5.37)	**3.40 (1.89–6.10)**	0.0001
Gestational age at delivery	Pre–term (<37wks)	43 (35.2)	79 (64.8)	1.58 (1.02–2.46)	1.15 (0.64–2.05)	0.648
	Term (≤ 37wks)	91 (25.6)	264 (74.4)	1	1	
Maternal blood loss	Yes	35 (61.4)	22 (38.6)	5.16 (2.89–9.20)	**4.78 (2.22–10.30)**	0.0001
	No	99 (23.6)	321 (76.4)	1	1	
Iron–folate supplementation	Yes	82 (23.3)	270 (76.7)	1	**1**	
	No	52 (41.6)	73 (58.4)	2.35 (1.52–3.62)	**3.27 (1.31–8.15)**	0.011
Pre–delivery Hgb status	<11.0 g/dl	63 (55.8)	50 (44.2)	5.30 (3.31–8.18)	**5.46 (3.09–9.67)**	0.0001
	**≥**11.0 g/dl	69 (19.2)	290 (80.8)	1	1	
Maternal MUAC measurement	**<**23 cm	36 (45.0)	44 (55.0)	2.50 (1.52–4.10)	1.52 (0.79–2.91)	0.694
	**≥**23 cm	98 (24.7)	299 (75.3)	1	1	

ANC, antenatal care; AOR, adjusted odds ratio; COR, crude odds ratio; Hgb, Hemoglobin; MUAC, mid-upper arm circumference; C/S, Ceaserean-section; SVD, spontaneous vaginal delivery; IAVD, instrumental assisted vaginal delivery; 1, reference category; IFA, Irona-folic acid. Bold values are those that were significantly associated with IPPA (outcome variable).

## Discussion

In this study, the overall magnitude of immediate postpartum anemia was 28.1%, 95% CI: (23.7–32.1%), which is comparable with findings from similar studies reported in Mbarara, Uganda (30.0%) (44); Coastal Karnataka (26.5%) (22); Madrid, Spain (29%) (27); Jimma, Southern Ethiopia (28.7) (30); Debre Markos, Northern Ethiopia (24.3) (24) and Mekelle (24.2) (21). However, the result of this study is encouraging as the current prevalence of immediate postpartum anemia is comparatively lower than the various previous studies conducted in Madrid, Spain (49.7%) (25); Beijing, China (32.7%) (45); Tamil Nadu, India (47.3%) (19); Bursa, Turkey (45.1%) (26) and Jeddah, Saudi Arabia (59.3%) (46). The possible explanation for this variation might be attributed to using different sub-standards of Hgb concentration cut-off points among the countries. Moreover, the selection of the study participants and assessment methods might be other factors for the discrepancy. For instance, Dundar and Cakmak selected women who had undergone episiotomy (26), and Rakesh et al. took Hgb < 12 g/dl at 6 weeks as a cut-off point, in contrast to this study (Hgb < 10 g/dl within 48 h of the postpartum period) (19). Moreover, in this study, we excluded women with certain preconditions such as diagnosed pre-conception anemia, uterine rupture, laparotomy, and multiple pregnancies to minimize the overestimation of postpartum anemia. Another possible explanation might be that the government is currently increasing the number of health extension workers in the rural community and has introduced a community health insurance program, motivating community members—including pregnant women—for health service utilization.

On the contrary, the number of postpartum anemia reported in the current study is higher when compared to the previous studies conducted in different parts of the world, like in Germany (22%) (47); Mariakani sub-country hospital, Kenya (16.4%) (48), and Ghana (16%) (20). The possible justification for these discrepancies might be attributed to ANC coverage, as can be seen in the studies by Bergmann et al., Rukiya et al. (48), and Kofie et al. (20), who selected only women who benefited from ANC follow-up. Besides, 65% of participants in the study undertaken in Ghana had completed secondary education (20) compared to only 23% of participants who had completed secondary and above education level in our study. Likewise, Kofie et al. and Rukiya et al. conducted a study at 6 weeks of the postpartum period, which is far from the immediate postpartum period (20, 48). As a result, the women may have recovered from anemia over time. Other possible reasons might be study settings, geographical differences, dietary habits, health-seeking behaviors, or different lifestyles of the community.

In the final model of the multivariable analysis, the educational status of the women was independently associated with immediate PPA. Accordingly, women without formal education had a significantly higher likelihood of developing anemia than women who had attended secondary and above-secondary education. This result is in agreement with a study conducted in Northern Ethiopia (21) and Nairobi, Kenya (48). This could be because education could allow mothers to use health care services, improve nutritional status by enhancing their preference for more diversified food, and make decisions for their health and their unborn fetus. Moreover, women's higher levels of education increase the use of ANC services, allow women more access to nutritional counseling during pregnancy and get the advantages of IFA supplementation (49). In other words, uneducated women have less access to maternal health care services such as ANC follow-up during pregnancy. Hence, they might not consume iron-rich nutritious food or make inappropriate use of IFA tablets during pregnancy, which is a pillar in the preventive strategies of anemia (29).

Likewise, the frequency of antenatal care visits was found to be an independent predictor of immediate PPA. Thus, women who had fewer than four ANC visits were more likely to develop immediate PPA when compared to those who had four or more ANC visits. This finding is in line with studies conducted in Debra Markos, Northern Ethiopia (24) and Jimma, Southern Ethiopia (30), and data from Ethiopia DHS (13), in which a higher number of anemia was observed in women with fewer ANC follow-ups. The possible justification could be that women who had < 4 ANC visits might not get enough provision of nutrition and health education adequately, and even if they do, they may not get enough provision of iron and folic acid, which contribute to the reduction of anemia and are not screened early for the detection of some diseases, including anemia and other complications that predispose to anemia. On the contrary, women who had adequate ANC visits benefited from prophylactic measures like malaria infection, monthly IFA supplementation, and early treatment of intestinal parasites (33, 40).

Moreover, in this study, the mode of delivery also increased the risk of being anemic during the postpartum period. Thus, the odds of developing immediate PPA increased among women who gave birth *via* cesarean section compared with those who gave birth *via* SVD. This finding is congruent with previous studies investigated in developing countries such as Southern Ethiopia (21), Jeddah, Saudi Arabia (46), and Pakistan (50) and developed countries like Madrid, Spain (27), and Bursa, Turkey (26). The possible explanation is that women who had undergone cesarean section might be predominantly susceptible to PPH, which, in turn, decreased RBC production and increased nutrient losses from bleeding (23). Another suggestion could be uterine atony from prolonged labor, uterine tears, lacerations from obstructed labor, and retro placental clot due to placenta abruption, which causes severe bleeding by preventing uterine contraction; they are all indications for cesarean delivery (51).

Similarly, the odds of immediate PPA among postnatal women with a history of blood loss during pregnancy or delivery were five times higher than those without a history of blood loss during the most recent pregnancy. Previous various studies also support this result report from California (23), Berlin, Germany (47), Tamil Nadu, India (19), and Debra Markos, Ethiopia (24). This is due to excessive bleeding at and/or after birth, which decreases the RBC component called Hgb. Moreover, hemoglobin values dropped by ≥2 mg/dL in women with 500–1,000 mL of blood loss (52). The other explanation might be the loss of iron stores during pregnancy, and blood loss during delivery could be the complications of antepartum bleeding.

Furthermore, hemoglobin levels less than eleven at delivery were another independent factor strongly associated with PPA during the immediate postpartum period. This finding is in line with different studies conducted in developed countries such as Berlin, Germany (47), Spain (25), California (23), and Tamil Nadu, India (19). The possible causes might be a low Hgb level before delivery and decreased myometrial contractility, as well as impaired coagulation, which results from impaired transport of Hgb and oxygen to the uterus, causing tissue enzymes and cellular dysfunction that lead to uterine atony, which is the most common cause of PPH (53).

Finally, women with no iron-folate supplementation during their most recent pregnancy were at risk of developing postpartum anemia. This finding is in agreement with studies from developing countries such as Debra Markos, Ethiopia (24), Karachi, Pakistan (50), and India (19). The possible explanation could be that iron is a necessary replacement for blood loss and tissue accretion during pregnancy and labor, as high physiologic requirements and depletion of iron during pregnancy and labor occur (54). Studies have implied that consumption of at least 90 iron-containing tablet supplements during pregnancy can reduce maternal anemia by up to 70%(55). Another justification might be that most women who are iron deficient but not anemic early in pregnancy become anemic due to decreased/ineffective/erythrocyte production, resulting in immediate PPA (56).

## Limitations of the study

As the women were asked about their activities in the past 10 months, recall bias was expected. However, best efforts were made to manage them through pre-testing questionnaires, training data collectors and supervisors on how to approach participants, interviewing postpartum women privately, close supervision of data collectors, and explaining the purpose of the study to the study participants well. Because the study was institutional-based, it may be difficult to generalize and apply the results to the general population. Moreover, as the study participants were from different geographical areas with different altitudes, adjusting Hgb levels based on altitudes was challenging. Additionally, this study also lacks an assessment of the dietary source of iron and the deworming status of the women during their recent pregnancy.

## Conclusion

This study indicated that the magnitude of immediate postpartum anemia is a moderate public health problem per the WHO cut-off value for the public health significance of anemia. About one-third of mothers admitted for postpartum care developed anemia within 48 h of giving birth. In this study, women's educational status, frequency of ANC visits, mode of delivery, maternal blood loss, pre-delivery hemoglobin concentration value, and iron and folic acid supplementation during recent pregnancy were independent factors associated with immediate PPA. This finding may help concerned bodies/stakeholders improve women's health through monitoring, implementing preventive measures, and sustained efforts on the identified risk factors of immediate postpartum anemia during pregnancy, labor, and delivery by understanding the local context of anemia in the early postpartum period.

## Data availability statement

The raw data supporting the conclusions of this article will be made available by the authors, without undue reservation.

## Ethics statement

The studies involving human participants were reviewed and approved by the Institutional Health Research Ethics Review Committee (IHRERC) of the College of Health and Medical Sciences, Haramaya University. Written informed consent to participate in this study was provided by the participants' legal guardian/next of kin.

## Author contributions

All authors listed have made a substantial, direct, and intellectual contribution to the work and approved it for publication.

## Conflict of interest

The authors declare that the research was conducted in the absence of any commercial or financial relationships that could be construed as a potential conflict of interest.

## Publisher's note

All claims expressed in this article are solely those of the authors and do not necessarily represent those of their affiliated organizations, or those of the publisher, the editors and the reviewers. Any product that may be evaluated in this article, or claim that may be made by its manufacturer, is not guaranteed or endorsed by the publisher.
